# Reframing conceptualizations of primary care involvement in opioid use disorder treatment

**DOI:** 10.1186/s12875-024-02607-x

**Published:** 2024-09-30

**Authors:** Kellia Chiu, Abhimanyu Sud

**Affiliations:** 1 Department of Family and Community Medicine, Temerty Faculty of Medicine, University of Toronto, Toronto, ON Canada; 2 School of Pharmacy, Faculty of Medicine and Health, The University of Sydney, Sydney, NSW Australia; 3 Humber River Health, Toronto, ON Canada

**Keywords:** Opioid-Related Disorders, Opiate Substitution Therapy, Health Systems, Primary Health Care

## Abstract

**Background:**

Opioid-related harms and opioid use disorder (OUD) are health priorities requiring urgent policy responses. There have been many calls for improved OUD care in primary care, as well as increasing involvement of primary care providers in countries like Canada and Australia, which have been experiencing high rates of opioid-related harms.

**Methods:**

Using Starfield’s 4Cs conceptualization of primary care functions, we examined how and why primary care systems may be suited towards, or pose challenges to providing OUD care, and identified health system opportunities to address these challenges. We conducted 14 semi-structured interviews with 16 key informants with experience in opioid use policy in Canada and Australia.

**Results:**

Primary care was identified to be an ideal setting for OUD care delivery due to its potential as the first point of contact in the health system; the opportunity to offer other health services to people with OUD; and the ability to coordinate care with other health providers (e.g. specialists, social workers) and thus also provide care continuity. However, challenges include a lack of resources and support for chronic disease management more broadly in primary care, and the prevailing model of OUD treatment, where addictions care is not seen as part of comprehensive primary care. Additionally, the highly regulated OUD policy landscape is also a barrier, manifesting as a ‘regulatory cascade’ in which restrictive oversight of OUD treatment passes from regulators to health providers to patients, normalizing the overly restrictive nature and inaccessibility of OUD care.

**Conclusions:**

While primary care is an essential arena for providing OUD care, existing sociocultural, political, health professional, and health system factors have led to the current model of care that limits primary care involvement. Addressing this may involve structurally embedding OUD care into primary care and strengthening primary care in general.

**Supplementary Information:**

The online version contains supplementary material available at 10.1186/s12875-024-02607-x.

## Background

Opioid use disorder (OUD) and opioid-related harms are significant public health concerns that require urgent policy attention. OUD is a chronic condition characterized by the persistent use of opioids despite adverse consequences [1]. It is a complex multifactorial condition involving interactions between individual- and structural-level factors such as stigma, discrimination, the drug supply environment, criminalization of drug use, and healthcare accessibility [2, 3]. Opioid agonist therapy (OAT) is an effective evidence-based treatment for OUD, reducing withdrawal symptoms and opioid-related harms such as toxicity and infectious disease transmission. OAT involves the structured use of an opioid agonist, typically methadone or buprenorphine [1]. 

Despite the effectiveness, there remains significant structural barriers and inequities for people with OUD in accessing OAT and subsequent treatment retention — previous research has estimated that fewer than 30% of people with OUD commence OAT, and even fewer remain in treatment beyond six months [4]. As part of the broad response to address increasing opioid-related harms and to improve OUD care, there have been many active calls for the improvement and better integration of OUD treatment in primary care [5, 6]. Primary care has been suggested as an optimal setting for OUD treatment delivery as it may help expand access to treatment and support the provision of comprehensive healthcare for people with OUD [7, 8].

OUD care can be accessed through primary care in numerous countries; for example, as ‘office-based’ buprenorphine treatment in the United States [9], methadone prescribed by general practitioners (GPs) with additional training in Ireland [10], or through primary care in conjunction with psychosocial care in Germany [11]. However, there are still many challenges to OAT access through primary care; for example, in Canada, there are limitations to treatment access for people living in rural or remote areas with fewer OAT providers and poorer access to primary care [12]. 

The delivery of OUD treatment in primary care can vary depending on political, sociocultural, geographical, and health system factors. Thus, improvement strategies require close examination of these factors and their influence on how and why current primary care systems may be conducive to or present barriers to providing OUD care.

In this analysis, we sought to understand from a health systems perspective how OUD treatment is provided in Canada and Australia through primary care, and the influential political, sociocultural, and geographical factors that have led to current delivery models. Both countries are currently experiencing high rates of opioid-related harms, and have developed and implemented OUD treatment within their health systems, including through primary care [6, 13, 14]. 

Using established frameworks describing primary care functions, we identified why and how current primary care systems may be conducive or present barriers to providing OUD care, and health system opportunities to address and improve these challenges. We also analyze current normative assumptions about OUD, its management (including through primary care), and existing paradigms of care.

### Research questions

1. How is OUD treatment currently delivered through primary care settings in Canada and Australia?
2. What are the factors that have influenced how OUD treatment is delivered through primary care?
3. What are the opportunities and barriers to improving the delivery of OUD treatment in primary care?

## Methods

### Study design

This study is a retrospective policy analysis, drawing on qualitative case study research methodology, using documentary analysis and semi-structured interviews, with hybrid deductive-inductive data analysis incorporating a theoretical framework (Starfield’s “4Cs” of primary care). This is an appropriate study design as we sought to understand existing interpretations and perceptions of OUD care in primary care, and how and why they have occurred. As this study is part of a broader body of work that seeks to identify policy lessons for Canada to improve OUD care, we chose a comparable country to analyze alongside Canada. In comparative health policy analyses, Australia is often used as a comparator to Canada due to their similarities regarding status as high-income countries, national health insurance systems [15], federal and decentralized governance structures, and the geographic distribution of the population [16]. The two countries are also experiencing significant levels of opioid-related harms, albeit with different epidemiologies. Therefore, the inclusion of both countries provides more depth to the analysis and opportunities for policy learning between two similar jurisdictions.

In Canada, the national rate of opioid overdose mortality is estimated at 20 per 100,000 population for 2022. This high rate is driven by the toxic unregulated drug supply and the prevalence of highly potent synthetic opioids like fentanyl primarily in the Western provinces and territories [17]. Provinces in which harms have been driven primarily by pharmaceutical opioids have much lower rates, and are comparable to the 3.8 opioid overdose deaths per 100,000 population in 2021 in Australia [18], which are also primarily due to pharmaceutical opioids [19]. In both countries, there has also been a push towards increasing and improving OUD care through primary care [6, 13]. Including both countries in our analysis provides opportunities for policy learning between the jurisdictions.

### Analytical framework

To explore the current delivery of OUD treatment in primary care, we used Starfield’s “4Cs” of primary care to guide our data coding and analysis.

Primary care has been recognised as an integral component of health systems, with more primary care-oriented health systems being associated with better health outcomes and more equitable distribution of health across populations [20]. To characterize the functions of primary care, Starfield describes primary care as comprising the following attributes: (1) *first contact*, where primary care is the first point of contact with the health system and health services; (2) *comprehensiveness*, where care is holistic and encompasses a range of health services; (3) *continuity*, where patients see the same healthcare professional, fostering a relationship over time; and (4) *coordination*, where primary care acts as the coordinating point for patients’ encounters with various parts of the health system [21]. 

This framework remains a widely used conceptualization of the role of primary care in health systems, seen, for example, in investigating interventions to enhance primary care [22, 23]. We identified the framework to be relevant for our study on OUD care in primary care as it allowed us to systematically examine from a health systems perspective the elements of primary care that may be conducive to providing OUD care, existing challenges inherent to current primary care, and strategies to address these challenges.

### Data sources and collection

#### Semi-structured interviews

Between February 2023 and June 2023, the lead author conducted 14 semi-structured interviews with 16 key informants who held relevant knowledge and experience on this topic. They were identified through purposive sampling based on a review of publicly available policy documents and our professional networks, and were contacted through email. Interviews lasted 25–94 min (average 52 min), and additional relevant potential participants were identified through snowball sampling until data saturation was reached. This occurred when the interview data that was collected contributed no new information and sufficiently demonstrated the application of the analytical framework to understanding the current delivery of OUD treatment in primary care.

Seven participants were from Canada and nine from Australia. Participants identified as persons with lived/living experience of drug use (*n* = 4); clinicians with experience working in the substance use disorder field (*n* = 9); researchers and academics (*n* = 7); staff of government health departments (*n* = 8); and individuals working in non-governmental organizations focusing on drug use (*n* = 7). Twelve participants identified as having multiple roles and perspectives. Table 1 presents a breakdown of participant roles by country. We also sent interview requests to 10 other individuals but received no response from six individuals and four individuals declined our invitation to participate.

**Table 1:** Participant roles by country

	Australia	Canada	Total
**Person with lived/living experience of drug use**	1	3	4
**Clinician**	5	4	9
**Researcher / academic**	4	3	7
**Staff of government health departments**	6	2	8
**Individuals working in non-governmental organizations focusing on drug use**	1	6	7

As our research questions focused on understanding the policy process and contextual factors influencing this, we developed an interview guide (Appendix I) informed by our analytical framework, as well as an established policy process heuristic [24]. It covered questions on participant’s opinions and experiences with OUD treatment; policy processes for drug policy, including influences on policymaking; and primary care and health system structures.

#### Documentary data

To provide context for the data collected through semi-structured interviews, we also collected data from documentary sources on each country’s health system and drug policy approaches, including treatment, harm reduction initiatives, and decriminalization [25]. For health system data, these documents included health system reports [26, 27] and websites published by government and health departments. For data on drug policy and OUD treatment, we collected national drug strategies and policies, data on drug use epidemiology and treatment, and clinical and operational guidelines for OUD treatment in each country between May 2022 and July 2023.

### Data analysis

Interviews were audio-recorded and transcribed; all interview transcripts and field notes were imported into the qualitative data analysis software, NVivo version 1.7.1 (QSR International).

We used a hybrid inductive-deductive approach to our qualitative data analysis [28]: we integrated a priori framework-driven categories, based on Starfield’s 4Cs, with inductively data-driven codes. This approach enabled the theoretical framework to be central to the analysis while allowing for themes to be generated using inductive coding. The lead author inductively coded interview transcripts into broad categories around health system, OUD treatment, and policy context (which included sociocultural, institutional policy process, geographic, regulatory, and political contexts) and reviewed with the second author. After discussion between both authors, these findings were then re-coded into each of the elements of the 4Cs framework. For each element of primary care, we described why it was considered important for OUD treatment in primary care; the current challenges and limitations, particularly regarding OUD treatment in primary care; and how these challenges could be addressed. In doing so, we analyzed the underlying health system, political, institutional, sociocultural factors that are driving these conceptualizations and challenges. Additionally, we identified another aspect of primary care not covered by the 4Cs (complexity) that appears to be pertinent to the discussion of better integrating OUD care into primary care.

### Research rigor and reflexivity

To ensure research rigor, the lead author took field notes during the interview process and wrote memos during data collection and analysis; these were continually re-visited throughout the research process for self-reflection. Data were coded by the lead author, in discussion with the second author and a broader research team (not directly involved in this project) to ensure validity in approach and findings.

The authors have experience with qualitative and quantitative research methods and have previously used these approaches to explore health policy and drug policy globally. Additionally, the authors are clinicians with clinical experience in community pharmacy in Australia (lead author) and family medicine in Canada (senior author).

## Results

We first describe the landscape of OUD treatment in primary care in both countries, based on documentary data. Following this, we present our semi-structured interview findings of the rationale, challenges, and enablers of OUD treatment in primary care.

### Summary of OUD treatment in primary care in Canada and Australia

In Australia, OUD treatment is available through public and private drug and alcohol clinics, as well as in the community through primary care practices and community pharmacies. As a federation, each subnational state/territory is responsible for its own organization of health workforce, funding of public hospitals and public health services, and OAT prescribing guidelines and regulations. As a result, there is cross-jurisdictional variability as to where patients are more likely to be able to access OAT [29]. The OAT medications available in Australia are oral methadone, sublingual buprenorphine and buprenorphine/naloxone in combination, and long-acting injectable buprenorphine. Prescribers (including nurse practitioners) typically need to undergo additional training and gain regulatory approval to prescribe OAT; similarly, pharmacies may also require approval to dispense OAT [29]. 

In Canada, OUD treatment can also be accessed through primary care physicians and community pharmacies, in addition to community-based outpatient clinics (e.g. low-barrier walk-in rapid access addiction medicine clinics [30]) and specialized treatment centers. Depending on the province/territory, nurse practitioners may also be able to prescribe methadone and buprenorphine after completing educational requirements [9]. Similar to Australia, exact delivery modes and treatment guidelines differ depending on the province/territory. Buprenorphine/naloxone is considered first-line therapy and generally has fewer physician prescribing requirements compared to methadone. Until May 2018, physicians required a federal exemption to prescribe methadone for OAT [31], and additional education/training, mentorship, or registration requirements [32]. Other OAT medications available include long-acting injectable buprenorphine, slow-release oral morphine, and injectable heroin and hydromorphone [33]. 

#### Differences between Canada and Australia

Australia has a high proportion of OAT treatment through the primary care system, in which services are delivered by private providers: in 2022, approximately 80% of all OAT prescribers were private prescribers (i.e. GPs), 62% of patients received OAT from a private prescriber [34], and 85% of dosing points were community pharmacies [35]. 

In Canada, there has been some data on the proportion of OAT delivery by different providers and in various settings. For example, in the province of Ontario, researchers have demonstrated that a majority of patients receive OAT from a small number of primary care-trained physicians who prescribe OAT in high-volume focused practice settings; these physicians tended to be older, male, and practice in urban settings [36]. In some provinces, regulatory college standards [37] suggest that treatment (particularly for methadone) be initiated in specialized clinics, with stabilized patients then maintained in community-based or primary care settings [12]. This appears to align with a generally stricter approach to the use of methadone for OAT compared to buprenorphine/naloxone, and calls to shift more OAT delivery to primary care.

Another difference between the two countries is the funding of OAT medications. In Australia, until recently, patients receiving OAT had their medications covered under a separate stream within the national medication subsidy program; however, patients receiving OAT from community pharmacies had to pay a dispensing fee which could total approximately $40AUD ($28USD) a week. From July 2023, the national program changed, where patients now pay a monthly co-payment (like with other medications), and the amount contributes to their overall yearly medication payment threshold. Additionally, this new policy forbids pharmacies from charging the additional out-of-pocket dispensing fee [38]. 

In contrast, coverage of the cost of OAT is regulated at the subnational level in Canada, where provinces set their own coverage rules. Depending on patient eligibility, the cost of OAT medications may be fully covered under provincial drug benefit plans (e.g. British Columbia [39]), or patients pay a co-payment that contributes to a deductible threshold (e.g. Saskatchewan [40], Ontario [41], Nova Scotia [42]).

### Analysis of OUD treatment in primary care

Based on our interview findings, Table 2 summarizes for each 4 C component the rationale behind improving OUD care in primary care, current challenges in primary care as it relates to OUD treatment, and potential strategies to address these challenges. This table was synthesized from participant interview data, as well as our own analysis on the underlying influential factors driving these conceptualizations.

**Table 2:** The rationale, challenges, and enablers of OUD care in primary care

	Why is this element important for OUD care in primary care?	What are the current challenges or limitations to having OUD care in primary care?	How could these challenges be addressed?
**First contact**	• Accessible healthcare • Primary care as the gateway to other services (including specialized substance use care or care relating to complications of substance use) • Primary care plays an important role in stewardship and prioritizing who needs access to sometimes limited addiction specialists and other health services, and who can be managed in primary care	• Ongoing access challenges to primary care • Primary care providers acting as gatekeepers may be a negative experience for people who use drugs, as they “control” access to further care and support • Primary care physicians may be reluctant to prescribe OAT • There may be regulatory caps on the number of patients for whom primary care physicians can prescribe OAT, and additional prescribing requirements	• Implement strategies to increase primary care access (through improving availability, accessibility, accommodation, affordability, acceptability) • Increase primary care physicians’ “competence and confidence” to manage OUD
**Continuity**	• A good ongoing relationship between a patient and their primary care providers can facilitate conversations about drug use • Community pharmacists are integral to care as patients typically see them more frequently than their physician	• Current conceptualization of continuity of OUD care in primary care can manifest in “locking in” patients to a specific prescriber and a specific community pharmacy for OUD treatment; this inflexibility is a challenge if patients need to access another provider (e.g. when providers are away/closed)	• Encourage more primary care physicians to take up prescribing OAT • Establish cross-coverage procedures for OAT prescribing and dispensing • Improve transitions of care pathways and liaisons between providers
**Comprehensiveness**	• OUD should be treated as a health issue like other chronic conditions • Patients with OUD may have interwoven health conditions • Patients should be able to get all their healthcare needs met in the same place	• Lack of funding, resources, and political support for comprehensive and chronic care in general • Current “two-tiered parallel” system for OUD treatment, where addiction services are not seen as part of comprehensive primary care	• Mechanisms to increase provider capacity (e.g. remuneration and incentives) • Exposure to substance use disorders (including OUD) as a means to address stigma and discrimination from healthcare professionals (e.g. through changes to medical school curricula) • Establish multidisciplinary clinics where patients can receive all their care in one place
**Coordination**	• For patients with OUD, other providers may be involved with care (e.g. social workers, nurses, pharmacists, addiction medicine specialists); primary care should be placed to coordinate these elements • Coordination supports comprehensive and continuous OUD care	• Some primary care physicians do not prescribe OAT, meaning patients may be receiving care from more than one physician; the lack of communication between providers can result in suboptimal care • Lack of referral pathway infrastructure and support for primary care providers in OUD care • Insufficient collaboration between different groups of healthcare providers (e.g. between physicians and pharmacists)	• Establish and improve referral pathways from primary to specialist care • Establish and formalize structures for coordinated multidisciplinary primary care • Improve data collection and sharing to plan care coordination (e.g. data on patient population, capacity of providers)

#### First contact

##### Rationale

One of the most prominent and commonly raised arguments for improving OUD care in primary care was its accessibility in the health system as the first contact point. In an ideal situation, this was envisioned as “making sure that everybody has a family doctor or nurse practitioner” and being able to access same day and after-hours care. Regardless of the complexity of patients’ health, this was acknowledged to be an essential service of any high functioning health system. As the first point of contact, primary care also serves as the gateway to other services (including specialized substance use care or care relating to complications of substance use).

Primary care often plays a gatekeeping role in the stewardship and prioritization of specialist and other health services, which may be a finite resource in the health system. One participant who had worked in a state health department described primary care’s gatekeeping role in the context of OUD care as “trying to make sure that those who need the most complex care can get it from an addiction specialty service. And those who don’t can be managed in primary care like any other mental health or any other kind of condition.”

##### Challenges and limitations

In theory, primary care as first contact is a benefit for OUD treatment because it means patients can have timely access to treatment with minimal barriers. However, there are a number of limitations to the accessibility of primary care in general, as well as barriers to having primary care as the first contact point for OUD care specifically.

Firstly, there are existing access challenges to primary care. Across both countries, participants pointed out that many people do not have a regular primary care physician and there are long waiting lists to join practices, where physicians may not be accepting new patients. In Australia, there is an additional barrier in that fewer primary care practices are “bulk-billing” i.e. more patients are having to pay a fee covering the gap between what the government covers and what a clinic charges for a GP consultation. In both countries, these challenges are also in conjunction with shortages in the primary care workforce, with older physicians retiring and fewer physicians entering primary care — a process that has been exacerbated by the many challenges of the COVID-19 pandemic. Therefore, for many people, their first contact with the health system may not be community primary care, but rather in other settings such as emergency departments.

Within this context, there are additional challenges — accessing primary care for people with OUD may be potentially stigmatizing. A participant with lived experience of drug use remarked that:

“[Primary care] really have control of so much — they have control of safe access, where it’s all through your medical system… they can be the difference between someone being able to make the phone call to access care and not. And they can also be the front door to the street.”

One participant, a primary care physician and researcher, noted that people with OUD were less likely to be able to be accepted into a primary care practice in the first place compared with patients with other chronic conditions, such as diabetes. This was thought to be due to stigmatizing views around drugs and the perception that people with OUD are more “complicated” for “regular primary care.”

OAT-specific regulations for prescribers and dispensers was identified as another limitation to having primary care providers as the first point of contact for people with OUD seeking treatment. This is seen through the caps on the number of patients for whom physicians can prescribe, and to whom pharmacies can dispense OAT medications. This is in conjunction with the additional requirements for prescribing OAT present in some subnational jurisdictions such as additional education/training, having to register with regulatory bodies, or being issued an authority to prescribe OAT.

Healthcare professional attitudes and values are also important factors. Acting as gatekeepers, primary care physicians were viewed by people who use drugs to “control” access to OAT and further support. Several government employees working in different Australian health departments noted that these attitudes of stigma and discrimination were embedded in the health system, and that health providers may often perceive people with OUD receiving treatment as “dangerous, risky, and untrustworthy.” This in turn affected decisions around where and how treatment is provided — particularly in primary care, physicians could choose not to prescribe OAT in their clinics, or pharmacies could choose not to dispense OAT, thus limiting access for patients.

#### Continuity

##### Rationale

Both clinicians and people who use drugs acknowledged that a good ongoing relationship between a patient and their primary care providers built trust and could facilitate conversations about drug use. Some participants also made specific reference to the role of community pharmacists in primary care, recognising their integral position in the health system as patients (especially those with chronic conditions requiring ongoing medications) typically interact with them more frequently than with physicians.

##### Challenges and limitations

Combined with the structurally stigmatizing attitudes from healthcare professionals and the health system, the current conceptualization and application of continuity of OUD care in primary care has manifested in ‘locking in’ patients to a specific prescriber and a specific community pharmacy for being prescribed and dispensed OAT. Indeed, this was highlighted by a few interviewees from Australia working in a regulatory capacity, who viewed these locked-in approaches as a means of *ensuring* continuity of care; however, this regulatory approach may also stem from the concern that these highly regulated OAT medications may be diverted, or that streamlined monitoring of people using drugs is required when being treated with OAT.

Although seeing the same primary care physician and pharmacist is conducive to developing a good relationship with healthcare professionals, this forced inflexibility was also identified to be another means of patients with OUD being “controlled” by the health system, and is also a challenge to receiving timely care if patients need to access another provider, for example, in situations when physicians are away, when pharmacies are closed, when traveling, or if patients have no fixed address.

This ‘locked in’ conceptualization of continuity may also pose challenges for OUD care accessibility. As we described previously, a number of Canadian participants noted that to improve OUD care in primary care, there needs to be a significant shift from the current parallel OUD treatment system of specialized addiction medicine and primary care clinics, to having most patients in the primary care system for treatment initiation, stabilization, and maintenance, preferably with their provider from whom they receive other care. For example, the Australian state of Victoria delivers OAT primarily through GPs and community pharmacies; while this may normalize OUD care in primary care, some Australian participants with drug policymaking experience at the state-level observed that better accessibility for people with OUD refers to more flexibility and choice, with a mix of treatment settings to suit people’s different needs.

Thus, this view of considering patient choice as an important element of care acts as a counterpoint to the regulatory drive to ‘lock in’ patients to particular treatment providers or settings, which may not serve all patients well — especially those at the margins, without fixed addresses or regular employment — who may not be able to see the same provider each time.

#### Comprehensiveness

##### Rationale

One of the most prominent arguments for the integration of OUD in primary care was the notion that it should be treated as a health issue, and particularly like other chronic conditions, given its chronic and relapsing nature. It was also argued that comprehensive primary care accounted for the fact that people with substance use disorders may often have interwoven concomitant health conditions and that “good substance use care involves good whole person care.” Therefore, moving OUD care “into more mainstream primary care” would provide a “one stop shop to more holistic care” — an opportunity to provide other healthcare, such as screening for cancer, or management of diabetes or cardiovascular disease.

A strategy to achieve this that was proposed by numerous participants was multidisciplinary team-based care, in which other providers such as nurses, social workers, addiction medicine specialists, and pharmacists would work together to ensure that the multifaceted care needs of people with OUD are comprehensively addressed, as they would be for the general population or people with other chronic diseases.

##### Challenges and limitations

A health system challenge to integrating better OUD care into primary care was the lack of funding, resources, and political support for comprehensive and chronic care in general. In terms of funding, interviewees — particularly those who were primary care physicians — commented that the current remuneration structures for delivering comprehensive care for chronic conditions were insufficient and did not adequately support physicians to provide this care. In Australia, a primary care physician and OAT prescriber remarked that:

“We have a Medicare system that is brilliant for self-limited, acute illness… it doesn’t work well for chronic care *in anything*. And there’s been tinkering at the edges with asthma management plans and team care arrangements and these kinds of things, but the bottom line is, it’s not fit for purpose anymore… there really does need to be a rethink around how we provide care for people with chronic complex issues, because at the moment, they’re missing out.”

Another challenge for comprehensive care was the apparent nascency of multidisciplinary team-based care, and the subsequent minimal funding and resources for it. Participants from both countries talked about the difficulties with establishing these structures, particularly in terms of the political support and sustained political commitment required to prioritize the funding and resourcing of multidisciplinary care in general.

“When things are externally funded, it’s actually really hard to get them designed from the ground up. They’re often governments coming in and wanting to do something in a very specific way… Allied health requires specific funding, and it was really hard to be able to get that and I think you really do need a lot of allied health support to get — you can’t just have physicians to run these good multidisciplinary clinics.” [primary care physician, Canada].”

This was similar to the situation in Australia, where pilot projects were implemented that brought together primary care physicians with specialist drug and alcohol services to deliver more comprehensive care for patients, but were lacking ongoing resources.

“We saw really good kinds of relationships put together there. But it was limited by funding; it started out really well with a lot of goodwill. But one of the things that happens, and we know this in general practice, is that it’s got to be sustained. Priorities change at a state and federal level. And they’re fairly short-term… And I think that is one of the misunderstandings that we really need to work really hard at with OAT is people do better if they’re in treatment long-term. This is a chronic illness, a chronic relapsing condition; it needs chronic continued funding. Not pilot programs that support GPs for a short period of time. Because it’s not like the patients get better and go away. They need that ongoing care.” [primary care physician and researcher, Australia].

Interviewees from both countries with clinical experience in OUD treatment also noted that there was often a lack of “ownership” and misconception from primary care physicians around providing OUD care, who may believe that treating people with OUD is out of their scope of regular practice, despite treating them for other health concerns.

“All people who come into contact with opioid use disorder clients, need to have some ownership. Yes, this is something that I could treat, and that could be seen within my practice. Otherwise, those patients are really made invisible, right? You hear people saying, “well, I don’t see people with opioid use disorder.” Well, no, you see them, it’s just that it’s not part of your practice to actually treat them. You might be admitting them for complications related to injection drug use, but you’re not treating the underlying condition.” [primary care physician and researcher, Canada].”

On a broader level, this segregation of OUD care from the wider health system was also reinforced by the interrelated sociocultural and political context. Treatment and harm reduction initiatives (such as supervised consumption sites) are all part of a comprehensive approach to OUD care; however, developing and implementing the appropriate facilities for this requires community support, which in turn, affect the priorities and political will (or lack thereof) of elected policymakers at the national and subnational level. In both countries, stigmatizing views from the public and community have been shown through the differing public perceptions around the use of different drugs and what is seen as ‘socially acceptable.’ Participants observed that there were “different responses to people who use opioids or heroin, versus those who use alcohol” and the wider acceptance of the latter in society. These entrenched views of opioid (and other illegal drug) use in society — particularly as an individual “moralistic failure” — is pervasive and impacts the political will of policymakers. Several interviewees with experience in policymaking for drug policy, or who had worked in close proximity to these policymakers, believed that policies came “from politicians and political war rooms where the goal is to win elections; none of it is based in evidence and it’s all based in who votes, who doesn’t, and who do we already stigmatize that we’re comfortable in continuing to stigmatize?” Thus, stigma and discrimination was identified to be a major contributing factor to the lack of resources, policy innovation, development, and implementation around OUD treatment as part of comprehensive primary care, and its current fringe status in healthcare.

#### Coordination

##### Rationale

Coordination is an important element of primary care that enables continuous and comprehensive care to be optimized. In the case of OUD treatment, other providers may be involved with care, including social workers, addiction medicine specialists, nurses, and pharmacists. Primary care is well-placed in the health system to coordinate these components.

We identified a number of coordination mechanisms across these two countries. In addition to the team-based structures discussed as part of primary care comprehensiveness, other mechanisms between primary care and specialist services included referral pathways, clinical liaisons, and specific care transition processes (e.g. switching prescribers).

##### Challenges and limitations

In the current context, there are challenges to continuity in primary care as many physicians do not prescribe OAT, resulting in patients receiving healthcare and services from more than one provider. This means that good communication (and infrastructure to support this) between providers is essential; if this is lacking, which some key informants with clinical experience observed it to be, this can result in suboptimal care.

Similar to the barriers with multidisciplinary team-based care, participants noted a lack of referral pathway infrastructure and support for primary care providers in OUD care. For example, interviewees with clinical experience contrasted the confidence and ability to refer patients with other health conditions to the appropriate specialist (e.g. endocrinologist for diabetes; orthopedic surgeon for advanced hip arthritis), to the lack of knowledge regarding how or who to refer to for substance use disorders. This was suggested to stem in part from relatively fewer addiction medicine specialists, as well as a lack of professional relationships with substance use care specialists.

Lastly, in addition to primary care having a coordinating role, it was also suggested that there needed to be system-level actors coordinating the health providers involved in OUD care. The state of Victoria in Australia operates a primary care-based OUD treatment model, where OAT is accessed almost exclusively via private GPs and community pharmacies. Reflecting on the Victorian context, one participant working in an Australian state department of health suggested that “you can’t have a bunch of prescribers just running around on their own and hope that everybody gets access to the services. There needs to be some kind of coordination.” This implied that while primary care may work as a coordinator to some degree, there was still a need to have strategic external coordination mechanisms for health providers involved in OUD treatment. In particular, several participants identified that “good data” on OUD prevalence and distribution and capacity of providers is needed for care coordination and planning.

#### Enablers of OUD care in primary care across the 4Cs

Enablers to improve OUD care in primary care can be broken down into strategies that address: (1) the broader health system challenges currently faced by primary care, and (2) more specific solutions for OUD treatment in primary care.

At the health system level, participants from both countries acknowledged that there needs to be significant primary care reform to improve primary care access in general: ensuring everyone in the population has their own primary care provider; establishing and investing in multidisciplinary team-based care structures, transitions of care pathways, and referral pathways and liaisons between providers (particularly primary and specialist care); and increasing remuneration and implementing appropriate financial incentives for primary care physicians and community pharmacists.

Many of the enablers that were proposed to increase and improve OUD care in primary care are aimed at ultimately supporting and increasing the number of primary care providers able to prescribe and dispense OAT. This would address some of the current challenges across the 4Cs: insufficiency of primary care as first contact for OUD care; lack of integration of OUD care in primary care; and patients receiving OUD treatment from different providers, reducing care continuity.

Participants suggested that to better address the reluctance of primary care providers to provide OAT, approaches to increase their “competence and confidence” as the first entry point for people with OUD, such as through increased education and establishing addiction clinics as core clinical placement opportunities during medical school and residency training. A Canadian drug policy researcher observed of the current situation that “it’s this weird dichotomy where they’re seen so much as the entry point and that first frontline where people can go to get help for any substance use concerns. But a message we keep hearing is just this issue of competence and confidence, like having the skills necessary to respond or the knowledge to know what to do to respond.”

Increasing exposure to substance use disorders (including OUD) was heavily emphasized as a means to address stigma and discrimination by healthcare professionals, with the goal of changing the current health professional and policy mindset that OUD care is separate from primary care. The normalization of OUD in clinical practice was seen to be critically important and many participants believed that this should be embedded as early as medical school, with changes to curricula to support this. After medical school, some participants also raised the possibility of physicians completing additional rotations in addiction medicine clinics; however, they acknowledged that this could send “mixed messages” as it “indicates that addiction again is something that is treated in a specialized clinic rather than in primary care.”

Similarly, strategies to encourage primary care physicians to take up OAT prescribing were also suggested as enablers to improve continuity for OUD care — this would ensure that patients would be able to see the same health providers for all their care, including the same physician who would prescribe OAT and manage treatment.

Given the current reality where patients may have more than one physician or pharmacy (i.e. one for OUD treatment, and another for other healthcare needs), solutions to improve communication were discussed. For example, a Canadian participant suggested the possibility of establishing centralized electronic databases that would contain patients’ medical records and prescriptions, allowing any provider to view these details. This would enable better information sharing between providers, but would also improve flexibility and accessibility for patients, allowing them to be able to present at any pharmacy for OAT.

#### Complexity as an additional element to the 4Cs

The notion of complexity was related to the rationale behind including OUD care as part of comprehensive primary care, but also appeared based on the assumption that primary care should be an ideal setting for managing patients who are stable on OAT and are “not that complex”, in contrast to “more complex cases” who would be treated in addiction specialty services. This stemmed from the view that patients with OUD and treatment with OAT are perhaps not as complex as understood or perceived to be, or that “basic OAT care” should be delivered in primary care, alongside other “basic care for people with opioid use disorder.”

The tension between viewing addiction medicine as complex care versus regular care, and how this influenced and continues to influence OAT delivery was consistently raised by clinician participants. The dominance of the view of addictions care as complex care has been facilitated by the development of OUD treatment settings, prescribing requirements, and education and training opportunities.

Historically, OUD treatment in both countries was set up in specialized settings, outside of primary care and considered a specialty or even sub-specialty, thus creating a parallel system and making it difficult to integrate into primary care.

“Right now, many stable patients are retained for years, and years, and years in these specialized clinics, when it would make far more sense that they’re getting that care in their primary care clinic. Then there’s more opportunities to treat them for other things, ensure they’re getting their appropriate screening, to have them connected to primary care.” [primary care physician and researcher, Canada].” All people who come into ;

Viewing addictions care as complex care rather than regular care suggests that it is too difficult and beyond the scope of primary care. Advocates for increased OUD treatment in primary care argue that it should be viewed as regular care with more complex patients referred to an addiction medicine specialist, like with other diseases such as diabetes or hypertension.

In addition to physicians, this conceptualization of OAT as an exception to regular care was also highlighted in the community pharmacy profession, who consider OAT as an “optional extra” with additional administrative burdens, and may choose not to stock and dispense methadone or buprenorphine, thus limiting where patients could receive their treatment. Several participants spoke of the need to reconsider whether OUD care is really as complex as it is made out to be, and the need to address the stigma, lack of confidence, and lack of support faced by primary care providers.

## Discussion

Primary care in Canada and Australia has the potential to better support care for people with OUD, as it is often the first point of contact with the health system, and has the capacity to provide holistic healthcare through offering comprehensive health services and coordinating with other parts of the health system. However, due to the historical and regulatory context of OAT; structural stigma permeating through societal, political, and health system landscapes; and lack of support and resources for primary care providers, OUD care has often been relegated to the “fringe” of healthcare. To address these challenges, many of the strategies suggested by interview participants involve increasing the confidence and competence of primary care physicians, and establishing better coordination pathways between providers, to ultimately increase the number of OAT prescribers.

Across our analysis, we also identified a few current conceptualizations of OUD treatment that may warrant further examination as to its influence on delivery through primary care: management of “complex cases” in specialist care, the consideration of OUD as a chronic disease, and the normalized higher level of regulation of opioid medications for OUD treatment.

### Complexity in OUD and primary care

We noticed that the use of ‘complexity’ in reference to OUD care seemed to refer to patients with ‘harder or more complicated’ cases of OUD that warranted the involvement of specialists. However, the concept of complexity in the context of opioid use has broader significance than this, pertaining to complexities in both health systems and patients. Scholars have previously defined the complexity of a system as the interrelatedness of system components, with increasing complexity as a result of many components with a high degree of interrelatedness between them [43]. This is particularly relevant in OUD care, where many providers and systems may be involved: primary care, including prescribers and pharmacy dispensing staff; medical specialists for addictions care and comorbid conditions; psychologists and counselors; and social workers. In complement, complexity can also apply to patients; this has been ill-defined, but previous research on patient complexity has identified it as involving medical aspects (e.g. high number of comorbidities, involving numerous body systems, taking multiple medications) or non-medical aspects (e.g. higher care coordination needs, socioeconomic disadvantage), or combinations of these, which may be present in people with OUD [44, 45]. 

One of the key competencies of family physicians, and advantages of the role within the health system, is their ability to manage and respond to intersecting, dynamic, and uncertain care complexities, including undifferentiated presentations, and non-medical patient and community challenges, such as efficiently mobilizing community resources to address needs relating to housing, employment, and family dynamics [46, 47]. Therefore, arguably, primary care could be a better and more appropriate venue through which to navigate and address the complexities inherent in OUD care. Although we have identified many challenges faced by primary care currently, the understanding of family physicians as being competent in managing complexity suggests that strengthening primary care is crucial to navigating health complexity more broadly.

### OUD as a chronic disease

Over the last few decades, a number of factors may have contributed to applying the chronic disease model to OUD: the growing understanding of the neurobiology and genetic underpinning of substance use disorders; the availability of effective medication-based treatment and their use for long-term management; the recognition of the need for sustained multidisciplinary support; and aspirations to “normalize” and destigmatize OUD and its care [48]. This was particularly evident in this study, where we noticed constant comparisons between OUD and diabetes, usually with the assertion that OUD should be treated as a chronic disease like diabetes. This aligns with current literature in which diabetes is often used as an exemplar of how a chronic disease care model can be applied [49–51]. 

Considering OUD as a chronic disease may be in line with normalizing and destigmatizing OUD; however, given the challenges with chronic disease management in general, there are opportunities to also see what other conceptualizations could offer to OUD care. For example, other scholars have proposed a ‘survivorship’ model; in contrast to viewing OUD as a chronic disease identity, this view involves thinking about OUD as similar to that of cancer, where individuals feel that they no longer have OUD, but their experience remains [52]. Further work is needed to fully explore the implications of a survivorship conceptualization on improving care — several of our participants noted that it could offer an avenue to garner more favorable political and societal attention, and challenge stigmatizing views; however, the authors found no significant differences in the general public between a chronic disease model and a survivorship model for reducing public stigma or increasing policy support [53]. 

Our findings also present an opportunity to question calls and strategies that simply graft OUD care onto current, poorly supported, and potentially unsustainable models of chronic disease care. While short-term strategies may be needed to address urgent drug-related harms, addressing the primary care needs of people with OUD is also an opportunity to re-think the delivery of primary care (including and beyond OUD care) from the perspective of people living at the margins. Indeed, some of the limitations of primary care for OUD treatment delivery that we identified through the 4Cs framework directly impact these populations. The current norms and characteristics of OUD care — including a lack of patient autonomy and flexibility, and a regulatory context that favors patients who have regular and unchanging health providers, fixed addresses, and regular employment — are indicative of an environment that may make it difficult for many people with OUD to access primary care and obtain necessary health care.

An alternative approach that has been implemented in Canada are low-threshold rapid access addiction medicine (RAAM) clinics, where individuals can access immediate OAT without needing an appointment or medical referral, and without needing to meet typical initiation procedures such as meeting strict eligibility criteria [54]. This more flexible model of care aims to provide patient-centered care that allows the patient to continue treatment at the clinic until they are ready to be transitioned to primary care for long-term management; additionally, these RAAM clinics can also connect patients to primary care and other health services [30, 55]. While these clinics are gaining traction in Canada (and are often staffed by clinicians with a primary care background), such models may not yet be sufficiently widespread, nor have they been implemented in Australia.

The importance of primary care to equitable health systems has often been touted, with international evidence from countries with primary care-oriented health systems demonstrating fewer inequities in health [56]. However, our findings demonstrate that in this current reality, even primary care remains inequitable for underserved people, with systems that do not adapt to their ongoing needs, barriers to accessing care, and insufficient funding and resourcing for programs [57]. A re-think of primary care to better deliver care for people who use drugs would involve embedding principles of justice, inclusion, and equity into any structural redesign of the health system, to prioritize and better center the needs of people at the margins first [58]. 

### Regulation of opioid agonist supply for OUD treatment

One of the noteworthy aspects of OUD treatment and OAT is its highly regulated nature and the mechanisms that maintain this — from our data, we identified a ‘regulatory cascade’ embedded throughout the care system, where stakeholders at all levels contribute to, or are subject to the inaccessibility of OUD care in primary care and “control” from upstream actors. At the health system level, the data collected on OAT prescriptions, and the number and locations of prescribers and dispensers are used to coordinate providers and patients and plan for service delivery. However, the monitoring function of data collection also serves to ensure that patients are only receiving OAT from their predetermined provider, and that providers are appropriate stewards of these highly regulated controlled substances. This cascade continues to manifest from the level of regulators to providers through additional requirements (e.g. accreditation, registration, and/or training), practice reviews or audits, or caps on the number of patients for whom providers can prescribe or dispense OAT [29, 32]. The strict regulatory cascade and control flows onto patients, in which they often need to present to a dosing point (e.g. community pharmacy) daily for witnessed dosing and ‘take-home’ doses are limited, patients are locked-in to specific prescribers and pharmacies, and they may be required to undergo urine drug screening [59]. 

This current restrictive regulatory and oversight paradigm appears normalized in both countries — as well as other international jurisdictions [25] — particularly in comparison to other clinical areas, and this may ultimately continue to reinforce the stigma towards people who use drugs, and the separation between OUD and other health conditions. To address this, the regulatory culture around OUD treatment needs to be reassessed, as well as strategies to actively target the structural stigma that leads to restrictive treatment policies [60]. 

### Strengths and limitations

Our analysis takes a novel approach to examining OUD treatment policy from a health systems perspective, using an established framework for understanding primary care. Starfield’s framework considers four key functions of primary care that enable it to provide efficient and effective care within the health system — using these key functions, this enabled us to explore from a health systems perspective how primary care has currently been conceived and positioned for delivering OUD care, as well as critically examine limitations tied to primary care delivery of OUD treatment, and identify opportunities to address these.

However, as our analysis has demonstrated, while the 4Cs framework encompasses important facets of primary care, it may be limited for capturing the complexities of health care for marginalized populations. For example, the function and rationale of continuity as it has been conceptualized — including assumptions and requirements that patients should attend the same prescribers and dispensing points — do not properly serve the needs of many people living at the margins. In the case of OUD, the broader regulatory, political, and sociocultural context of opioid use has led to a treatment model involving primary care with regulatory priorities that conflict with the priorities, needs, and wellbeing of many people who use drugs.

In terms of other limitations, most participants were currently primarily working in more populous and resourced areas of both countries. However, participants provided a range of experiences and insights, with many of them working at a national level or having experience working in rural, remote, Indigenous, and culturally and linguistically diverse communities, so were able to provide a more holistic view of OUD care in various primary care contexts.

Both countries are federal systems with subnational policy actors responsible for many aspects of OUD treatment; therefore, there may be within-country variation regarding care delivery. As such, it may be difficult to represent and compare ‘national’ models of care; however, our participants identified common themes applicable to the whole country, and between countries, which we analyzed and reported. Additionally, as this study was not specifically an analysis of subnational jurisdictions, future research should closely compare within-country OUD treatment policies.

Our analysis was limited to Australia and Canada; however, there may be policy lessons for similar high-income jurisdictions to learn from other countries who also deliver OAT through primary care. France is often seen as an example, having deregulated buprenorphine prescribing so that it can be prescribed by any physician, with training encouraged but not mandatory. Additionally, treatment is delivered through multidisciplinary teams (GPs, social workers, psychologists, and specialists) that have been formalized for substance use disorder care, and are distributed across the country; these structures are undergoing efforts to be extended into general chronic disease management [61]. Given that both Canada and Australia have goals of increasing multidisciplinary team-based care in both substance use disorder and chronic disease care more broadly [8, 62–64], France may be a comparable example to further analyze and understand how their policy context may be conducive to this strategy.

## Conclusions

While primary care remains an important venue through which accessible OUD care should be provided in Canada and Australia, there are current challenges to OUD care delivery that extends beyond the nature of treatment itself to the broader limitations currently faced by primary care. Through understanding the various conceptualizations of care within existing health system, political, and regulatory environments, our analysis suggests that there are multiple aspects of care delivery that may benefit from reconsideration, from the perception of OUD as a chronic disease, to challenging the assumptions and norms behind the restrictive regulatory nature of OAT provision.

## Electronic supplementary material

Below is the link to the electronic supplementary material.

Supplementary Material 1

## Data Availability

The COnsolidated criteria for REporting Qualitative research (COREQ) checklist, semi-structured interview guide, and data analysis codebook are included in the following Open Science Framework project repository: https://osf.io/cjfk3/?view_only=a025b21121ed46e09e7e9a95c6aa0d17.
