# Supplementary material for: Reframing conceptualizations of primary care involvement in opioid use disorder treatment
Source: BMC Prim Care. 2024 Sep 30;25:356. doi: 10.1186/s12875-024-02607-x (PMC11443781; doi:10.1186/s12875-024-02607-x)
Supplement: Supplementary file 1 — Supplementary Material 1 [file 12875_2024_2607_MOESM1_ESM.docx]

**Appendix I. Semi-structured interview guide**

| **General topic** | **Questions / Prompts** |
| --- | --- |
| Background / about yourself | - What might be important for me to know about you, in order to understand what you may say in the interview - What do you think about the state of opioid use disorder treatment in your country?   - About the role of opioid agonist therapy? - What do you think about other measures used to address OUD / opioid crisis / drug poisoning? |
| Your experience | - How have you been involved in this area of policymaking? - What part of the process were you involved in? (e.g. initial discussion, agenda setting, formulating policy, implementation, evaluation) - What were your responsibilities? |
| Policy development process | - What do you think were/are the key factors that have led to your country’s current OUD care model?   - Health system   - Drug use epidemiology   - Sociocultural (including values)   - Political / institutional   - Geographical - How has this context shaped policies and clinical practice? (and ultimately, health outcomes) - Who are the key policy actors / stakeholders?   - From your perspective, who were/are the most influential? Why? - What were the strengths of the policy development process? - What were the challenges and limitations of the policy development process? - What strategies do you think can be used to improve on any limitations, particularly for future policy development? - What kinds of evidence or knowledge was considered? How was it used? |
| Context and policy | - [see above section] - Intersection between existing OUD treatment policies, clinical practice, clinical education   - How do these work / not work together?   - How do these pose structural challenges or benefits to OUD care? |
| Opioid use in primary care | - What do you think about the role of primary care in opioid use care? - What prompted the involvement of primary care in OUD care? - How does primary care structurally facilitate or hinder opioid use care? - How could the role of primary care be changed to meet healthcare / OUD care needs? |
| Wrap-up / admin | - Is there anything else that you think is important for me to know, with regards to the aims of our study? - Would you be willing to participate in a follow-up interview? |
